# HIV-1 Nef synergizes with APOL1-G1 to induce nephrocyte cell death in HIV-related kidney diseases

**DOI:** 10.1242/dmm.052178

**Published:** 2025-08-01

**Authors:** Jun-yi Zhu, Yulong Fu, Joyce van de Leemput, Jing Yu, Jinliang Li, Patricio E. Ray, Zhe Han

**Affiliations:** ^1^ Center for Precision Disease Modeling, Department of Medicine, University of Maryland School of Medicine, Baltimore, MD 21201, USA; ^2^ Division of Endocrinology, Diabetes and Nutrition, Department of Medicine, University of Maryland School of Medicine, Baltimore, MD 21201, USA; ^3^ Child Health Research Center, Department of Pediatrics, University of Virginia School of Medicine, Charlottesville, VA 22903, USA; ^4^ Children's Research Institute, Children's National Health System, Washington, DC 20012, USA

**Keywords:** APOL1, HIV, Nephropathy, Nef, Nephrocyte, Autophagy, HIVAN, *Drosophila*

## Abstract

People carrying two *APOL1* risk alleles (RA) – *G1* or *G2* – are at greater risk of developing human immunodeficiency virus (HIV)-associated nephropathy (HIVAN). However, it remains unclear whether the encoded protein(s) (APOL1-RA) and HIV-1 Nef interact to induce podocyte cell death. Here, we generated transgenic flies that express *APOL1-G1* (derived from a child with HIVAN) and HIV-1 *nef* specifically in the nephrocytes, the fly equivalent of mammalian podocytes, and assessed their individual and combined effects on the nephrocyte filtration structure and function. We found that HIV-1 Nef acts in synergy with APOL1-G1, resulting in nephrocyte structural and functional defects, and that Nef exacerbates the organelle acidification defects and autophagy reduction induced by APOL1-G1. The synergy between HIV-1 Nef and APOL1-G1 is built on their joint effects on elevating endoplasmic reticulum (ER) stress, triggering nephrocyte dysfunction and, ultimately, cell death. Thus, we identified ER stress as the converging point for the synergy between HIV-1 Nef and APOL1-G1 in inducing nephrocyte cell death. Given the high similarity between *Drosophila* nephrocytes and human podocytes, our findings suggest ER stress as a new therapeutic target for HIV-1- and APOL1-associated nephropathies.

## Teaser

**Summary:** In a new *Drosophila* model, we identified endoplasmic reticulum stress as the converging point for synergy between APOL1-G1 and HIV-1 Nef in kidney cells, providing a potential therapeutic target for HIV-1- and APOL1-associated nephropathies.

## INTRODUCTION

Human immunodeficiency virus (HIV)-associated nephropathy (HIVAN) is a kidney disease characterized by heavy proteinuria and rapid progression to chronic kidney failure (Ross, 2014; Swanepoel et al., 2018). HIVAN renal histology shows collapsing glomerulopathy, de-differentiation and proliferation of podocytes and glomerular parietal epithelial cells, development of focal and segmental glomerulosclerosis (FSGS), and microcystic dilatation of renal tubules, leading to kidney enlargement (Ross, 2014; Swanepoel et al., 2018). People of African descent are at increased risk of developing HIVAN. Their risk to develop HIVAN or another chronic kidney disease is strongly associated with two apolipoprotein L1 (*APOL1*) risk alleles (*APOL1-RA*), *G1* and *G2* (Kopp et al., 2011; Kasembeli et al., 2015). People living with HIV-1 that carry two copies of the *APOL1-RA* have a ∼50% lifetime risk of developing HIVAN (Kopp et al., 2011; Kasembeli et al., 2015; Rednor and Ross, 2018), and poorly controlled HIV-1 infection is the most powerful factor known to contribute to APOL1-associated kidney diseases (Kasembeli et al., 2015). However, to date, the basic mechanisms through which the APOL1-RA interact with HIV-1 to cause HIVAN remain unclear.

Mice and rats do not have an *APOL1* ortholog, nor can they be infected with HIV-1 (Keppler et al., 2002; Baumann et al., 2004). Nonetheless, transgenic (Tg) rodent models have been used to characterize the molecular mechanism through which the *APOL1-RA* and HIV-1 genes induce renal diseases (Li et al., 2017; Yoshida et al., 2023). HIV-Tg26 mice and rats carrying a replication-defective HIV-1 construct (driven by HIV long terminal repeat, lacking a 3 kb sequence with the *gag* and *pol* genes, which are essential for viral replication), showed that the expression of HIV genes in podocytes and tubular epithelial cells plays a critical role in inducing HIVAN in rodents (Kajiyama et al., 2000; Ray et al., 2003; Zuo et al., 2006; Lu et al., 2008; Xie et al., 2014; Hu et al., 2023). In addition, several HIV-Tg mouse models showed that the HIV accessory protein negative factor (Nef), is a key determinant of HIVAN pathogenesis (Kajiyama et al., 2000; Ray et al., 2003; Zuo et al., 2006; Lu et al., 2008; Xie et al., 2014; Hu et al., 2023). Studies in cultured kidney cells and *APOL1* Tg mice found that the expression levels and localization of APOL1 are crucial in determining its cytotoxicity (Fu et al., 2017a; O'Toole et al., 2018; McCarthy et al., 2021; Bruggeman et al., 2019). Notably, different dual APOL1-HIV-Tg26 mouse models have generated conflicting results, supporting either toxicity or protection (Fu et al., 2017a; Zhang et al., 2013). Thus, it remains unclear whether HIV-1 Nef and APOL1-RA interact directly to induce HIVAN, underlining the need for new animal models.

*Drosophila* is an established model for kidney diseases and development (O'Toole et al., 2018; Fu et al., 2017b; Hermle et al., 2017; Kruzel-Davila et al., 2017; Gerstner et al., 2022; Rani and Gautam, 2018; Weavers et al., 2009). The fly nephrocyte is equivalent to the mammalian podocyte, with a highly conserved filtration structure called the slit diaphragm (Zhuang et al., 2009; Wang et al., 2021; Lang et al., 2022; Zhu et al., 2023). They also share many other similarities in endocytosis and exocytosis for the formation and maintenance of the filtration structure (Zhuang et al., 2009; Wang et al., 2021; Lang et al., 2022; Zhu et al., 2023). A majority of genes associated with kidney diseases have functional homologs in flies that are required for nephrocyte function (Hermle et al., 2017; Weavers et al., 2009). The *Drosophila* nephrocyte has been well established to study the pathogenesis of *APOL1*-associated nephropathies (O'Toole et al., 2018; Gerstner et al., 2022; Rani and Gautam, 2018; Lee et al., 2023; Han and Olson, 2005). Here, we generated Tg flies that express HIV-1 *nef* and *APOL1-G1*, the most frequent risk variant, specifically in nephrocytes. Our findings indicate that Nef and APOL1-G1 act synergistically to induce nephrocyte cell death through the endoplasmic reticulum (ER) stress pathway, providing a potential therapeutic target for HIV-1- and APOL1-associated nephropathies.

## RESULTS

### HIV-1 Nef exacerbates APOL1-G1-induced nephrocyte dysfunction

We previously generated Tg *Drosophila* lines with nephrocyte-specific expression of *APOL1-G0* or the *APOL1-G1* risk allele derived from a child with HIVAN (O'Toole et al., 2018). Here, we generated Tg flies with nephrocyte-specific expression of HIV-1 *nef* to explore how it alone or combined with *APOL1* affects the structure and function of nephrocytes. In our previous studies (Fu et al., 2017a; Lee et al., 2023), we used female flies to demonstrate that APOL1 toxicity is dose dependent. At 22°C, *APOL1-G0* flies showed no detectable phenotype at days 1 and 20, whereas *APOL1-G1* flies exhibited significant toxicity as early as day 1, which became even more pronounced by day 20. Based on these findings, we selected 20-day-old female flies for this study to maintain consistency with our previous results.

**Fig. 1.**
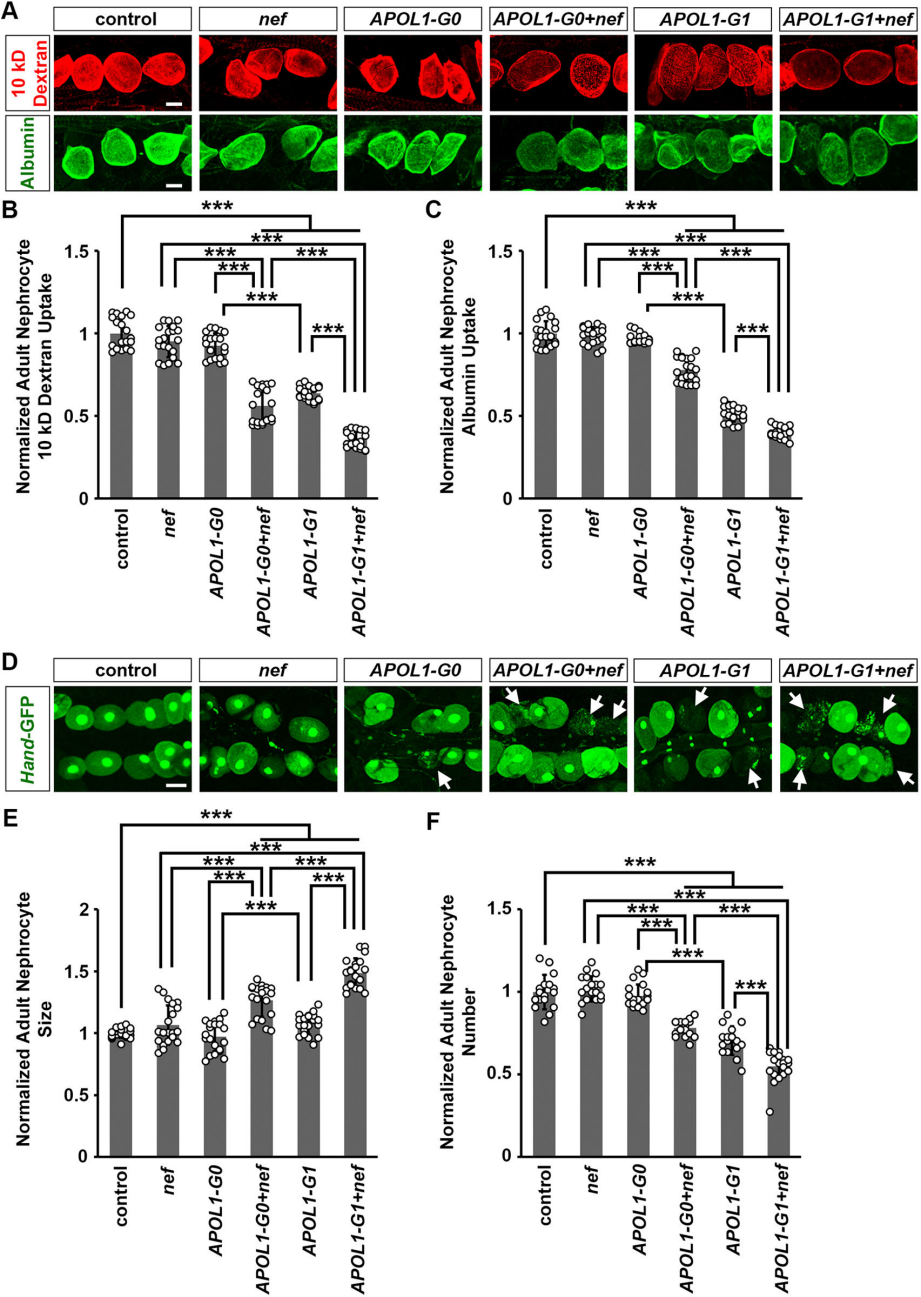
**Expression of HIV-1 protein Nef facilitated nephrocyte function reduction, hypertrophy and cell death due to APOL1-G1.** Flies used (20-day-old adult females): control (*Dot*>*w*^11^18); *nef* [*Dot*>*nef*-overexpression (OE)]; *APOL1-G0* (*Dot*>*APOL1-G0*-OE); *APOL1-G1* (*Dot*>*APOL1-G1*-OE); *APOL1-G0+nef* (*Dot*>*APOL1-G0*-OE+*nef*-OE); *APOL1-G1+nef* (*Dot*>*APOL1-G1*-OE+*nef*-OE). (A) Top: 10 kDa fluorescent dextran particle uptake (red) by nephrocytes using the nephrocyte-specific driver *Dot*-Gal4 to express HIV-1 *nef* alone or together with *APOL1-G0* and *APOL1-G1* at 22°C. Bottom: FITC-albumin particle uptake (green) by nephrocytes using the nephrocyte-specific driver *Dot*-Gal4 to express HIV-1 *nef* alone or together with *APOL1-G0* and *APOL1-G1* at 22°C. Scale bars: 15 µm. (B) Quantitation of 10 kDa dextran uptake, relative to uptake in control flies. *n*=20 flies, per group. (C) Quantitation of FITC-albumin uptake, relative to uptake in control flies. *n*=20 flies, per group. (D) Representative images of nephrocytes using the nephrocyte-specific driver *Dot*-Gal4 to express HIV-1 *nef* alone or together with *APOL1-G0* and *APOL1-G1* at 22°C. Scale bar: 25 µm. (E) Quantitation of adult nephrocyte size, relative to size in control flies. *n*=20 flies, per group. (F) Quantitation of adult nephrocyte number, relative to cell number in control flies. *n*=20 flies, per group. Results are presented as mean±s.d., normalized to the control group. Kruskal–Wallis H-test followed by a Dunn's test; ***P<0.001.

We determined the capacity of dissected nephrocytes to filter and endocytose 10 kDa dextran or larger FITC-albumin particles. Neither HIV-1 *nef*- nor *APOL1-G0*-expressing nephrocytes showed a reduction in uptake of dextran or FITC-albumin (Fig. 1A-C). Nephrocytes expressing *APOL1-G0*+*nef* or *APOL1-G1*+*nef* showed significantly decreased uptake of dextran and FITC-albumin compared to those expressing *nef*, *APOL1-G0* or *APOL1-G1*, with the greatest reduction in *APOL1-G1*+*nef*-expressing nephrocytes (Fig. 1A-C), reflecting the higher toxicity of the *APOL1-G1* risk allele. These results show that HIV-1 Nef exacerbates APOL1-induced nephrocyte dysfunction.

### Combined HIV-1 Nef and APOL1 cause nephrocyte hypertrophy and cell death

Previously, we showed that *APOL1-G1* induces progressive hypertrophy and accelerated cell death in nephrocytes (O'Toole et al., 2018). Hypertrophy was validated here in 20-day-old fly nephrocytes expressing *APOL-G1* (Fig. 1D,E). Nephrocytes expressing HIV-1 *nef* or *APOL1-G0* did not show a significant change in size, whereas those expressing *APOL1-G0*+*nef* or *APOL1-G1*+*nef* were significantly larger, with *APOL1G1*+*nef* nephrocytes the largest (Fig. 1E).

We observed reduced nephrocyte numbers in 20-day-old *APOL1-G1* Tg flies (Fig. 1D,F). However, we did not observe significant changes in nephrocyte numbers in flies expressing HIV-1 *nef* or *APOL1-G0* (Fig. 1F). In contrast, flies expressing *APOL1-G0*+*nef* or *APOL1-G1*+*nef* showed a significant reduction in nephrocyte number compared to those expressing *APOL1-G0* or *APOL1-G1*, with numbers lowest in flies expressing *APOL1-G1*+*nef* (Fig. 1F). These results show that HIV-1 Nef combined with APOL1-G1 exacerbates the damage to and loss of nephrocytes.

### HIV-1 Nef and APOL1-G1 combined disrupt the slit diaphragm filtration structure

**Fig. 2.**
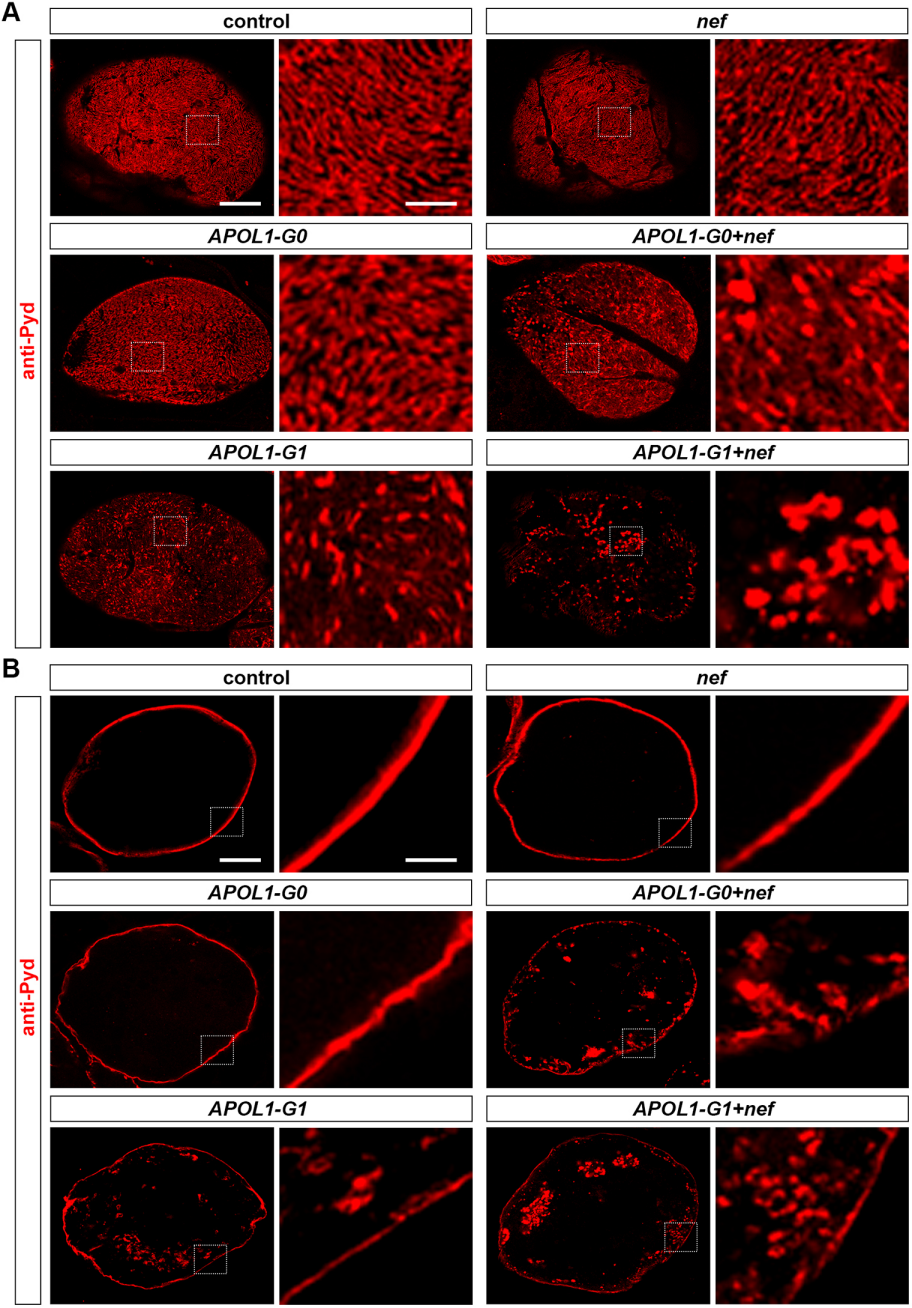
**Expression of HIV-1 protein Nef in nephrocytes facilitated disruption of the slit diaphragm due to APOL1-G1.** Flies used (20-day-old adult females): control (*Dot*>*w*^1118^); *nef* (*Dot*>*nef*-OE); *APOL1-G0* (*Dot*>*APOL1-G0*-OE); *APOL1-G1* (*Dot*>*APOL1-G1*-OE); *APOL1-G0+nef* (*Dot*>*APOL1-G0*-OE+*nef*-OE); *APOL1-G1+nef* (*Dot*>*APOL1-G1*-OE+*nef*-OE). (A,B) Localization of the slit diaphragm protein Polychaetoid (Pyd; red) in surface (A) and medial (B) optical sections in nephrocytes using the nephrocyte-specific driver *Dot*-Gal4 to express HIV-1 *nef* alone or together with *APOL1-G0* and *APOL1-G1* at 22°C. Boxed areas are shown magnified next to each image. Scale bars: 5 μm; 1 μm (magnified).

The *Drosophila* slit diaphragm is a highly organized structure critical for nephrocyte filtration (Zhuang et al., 2009). Thus, we assessed the localization of the slit diaphragm protein Polychaetoid [Pyd; homolog of human tight junction protein ZO-1 (also known as TJP1)] (Lang et al., 2022). Control nephrocytes showed the typical fingerprint-like localization pattern of Pyd on the cell surface (Fig. 2A) and the characteristic continuous circular ring in the optical medial view (Fig. 2B). Notably, nephrocytes expressing *nef* or *APOL1-G0* did not show visible disruption of Pyd (Fig. 2A,B). However, nephrocytes expressing *APOL1-G0*+*nef* or *APOL1-G1*+*nef* showed highly disorganized Pyd localization, more so than for those expressing either *APOL1* risk variant alone. Moreover, mislocalized Pyd was most evident in nephrocytes that expressed *APOL1-G1*+*nef* (Fig. 2A,B), consistent with the altered uptake observed in nephrocytes expressing *nef* and *APOL1-G1* (Fig. 1), which indicates a disrupted slit diaphragm filtration structure.

### Combined HIV-1 Nef and APOL1-G1 exert detrimental effects on nephrocyte endocytosis

**Fig. 3.**
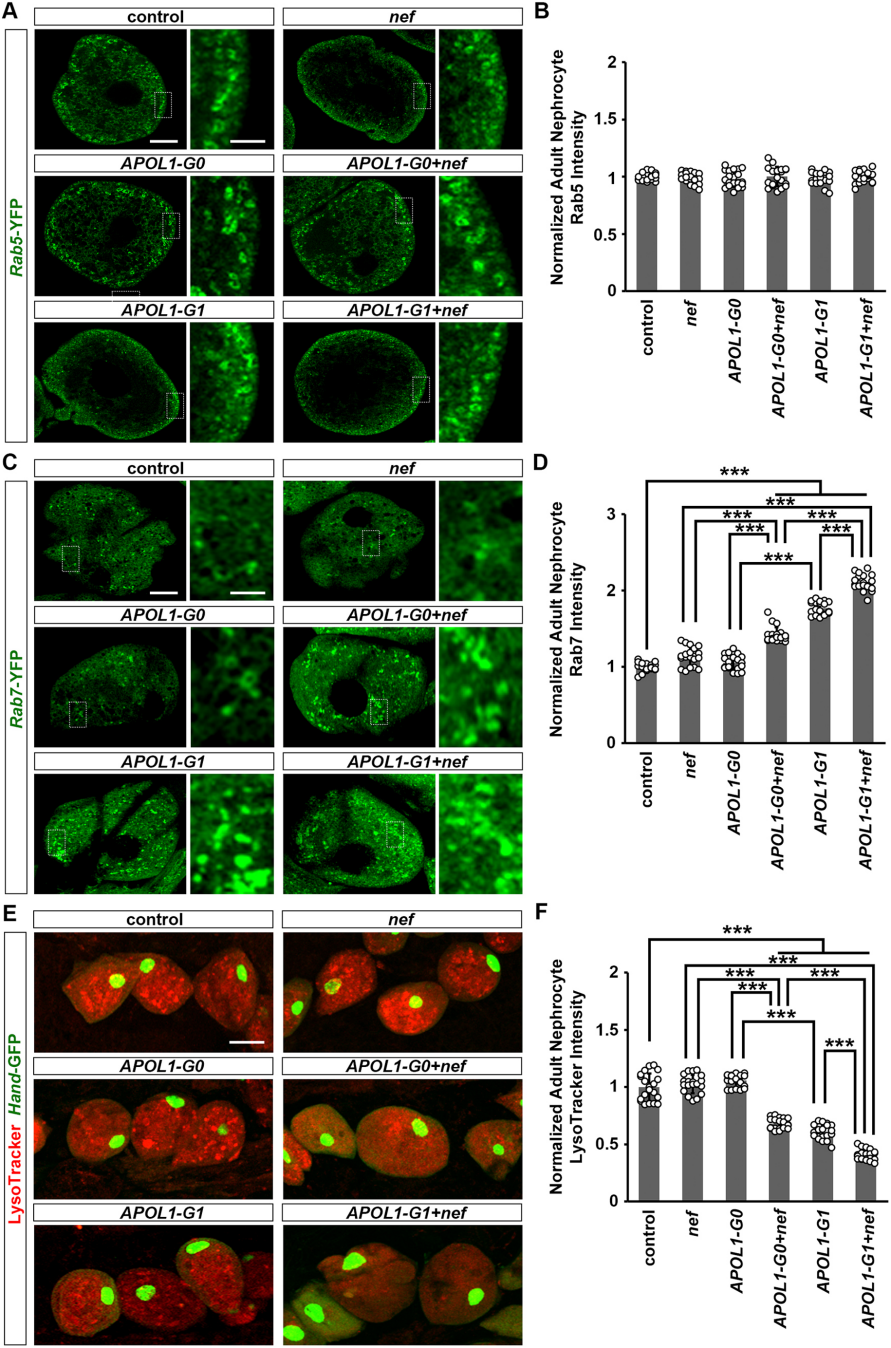
**Expression of HIV-1 protein Nef in nephrocytes facilitated the disruption in endocytic membrane trafficking due to APOL1-G1.** (A,B) Flies used (20-day-old adult females): control (*Dot*>*Rab5*-YFP); *nef* (*Dot*>*Rab5*-YFP *+nef*-OE); *APOL1-G0* (*Dot*>*Rab5*-YFP*+APOL1-G0*-OE); *APOL1-G1* (*Dot*>*Rab5*-YFP*+APOL1-G1*-OE); *APOL1-G0+nef* (*Dot*>*Rab5*-YFP*+APOL1-G0*-OE+*nef*-OE); *APOL1-G1+nef* (*Dot*>*Rab5*-YFP*+APOL1-G1*-OE+*nef*-OE). (C,D) Flies used (20-day-old adult females): control (*Dot*>*Rab7*-YFP); *nef* (*Dot*>*Rab7*-YFP *+nef*-OE); *APOL1-G0* (*Dot*>*Rab7*-YFP*+APOL1-G0*-OE); *APOL1-G1* (*Dot*>*Rab7*-YFP*+APOL1-G1*-OE); *APOL1-G0+nef* (*Dot*>*Rab7*-YFP*+APOL1-G0*-OE+*nef*-OE); *APOL1-G1+nef* (*Dot*>*Rab7*-YFP*+APOL1-G1*-OE+*nef*-OE). (E,F) Flies used (20-day-old adult females): control (*Hand*-GFP, *Dot*>GFP+*w^1118^*); *nef* (*Hand*-GFP, *Dot*>*nef*-OE); *APOL1-G0* (*Hand*-GFP, *Dot*>*APOL1-G0*-OE); *APOL1-G1* (*Hand*-GFP, *Dot*>*APOL1-G1*-OE); *APOL1-G0+nef* (*Hand*-GFP, *Dot*>*APOL1-G0*-OE+*nef*-OE); *APOL1-G1+nef* (*Hand*-GFP, *Dot*>*APOL1-G1*-OE+*nef*-OE). (A) Expression of endocytosis-related protein Rab5 (green, early endosome) in nephrocytes using the nephrocyte-specific driver *Dot*-Gal4 to express HIV-1 *nef* alone or together with *APOL1-G0* and *APOL1-G1* at 22°C. Boxed areas are shown magnified next to each image. Scale bars: 5 μm; 1 μm (magnified). (B) Quantitation of Rab5 fluorescence intensity, relative to fluorescence in control nephrocytes. *n*=20 flies, per group. None of the comparisons between groups were significant. (C) Expression of endocytosis-related protein Rab7 (green, late endosome) in nephrocytes using the nephrocyte-specific driver *Dot*-Gal4 to express HIV-1 *nef* alone or together with *APOL1-G0* and *APOL1-G1* at 22°C. Boxed areas are shown magnified next to each image. Scale bars: 5 μm; 1 μm (magnified). (D) Quantitation of Rab7 fluorescence intensity, relative to fluorescence in control nephrocytes. *n*=20 flies, per group. (E) LysoTracker dye fluorescence level (red) in nephrocytes using the nephrocyte-specific driver *Dot*-Gal4 to express HIV-1 *nef* alone or together with *APOL1-G0* and *APOL1-G1* at 22°C. *Hand*-GFP transgene expression was visualized as green fluorescence concentrated in the nuclei of nephrocytes. Scale bar: 15 μm. (F) Quantitation of LysoTracker dye fluorescence intensity, relative to fluorescence in control nephrocytes. *n*=20 flies, per group. Results are presented as mean±s.d., normalized to the control group. Kruskal–Wallis H-test followed by a Dunn's test; ***P<0.001.

We previously showed that HIV-1 can infect cultured human podocytes via dynamin-dependent endocytosis in a process facilitated by transmembrane TNF-α (also known as TNF) (Li et al., 2017). Here, we assayed the expression of early (Rab5) and late (Rab7) endosomal markers in *Drosophila* nephrocytes. In *Drosophila* nephrocytes, Rab7 puncta can appear as circles in normal conditions. Neither expression of *nef*, *APOL1-G0* or *APOL1-G1*, nor their combination, induced changes in the expression level or localization of Rab5 (Fig. 3A,B). In contrast, Rab7 protein levels were significantly increased in nephrocytes expressing *APOL1-G1*, but not in those expressing *nef* or *APOL1-G0* (Fig. 3C,D). Nephrocytes expressing *APOL1-G0*+*nef* or *APOL1-G1*+*nef* had significantly higher Rab7 protein levels, with the highest expression levels in *APOL1-G1*+*nef* nephrocytes (Fig. 3C,D).

We also showed previously that *APOL1-G1* altered the acidification of organelles and lysosomes in nephrocytes, which could impair their function (O'Toole et al., 2018). Here, we used LysoTracker to examine acidic vacuoles and lysosomes in nephrocytes with *nef* and *APOL1* expression. LysoTracker signal was significantly reduced in nephrocytes expressing *APOL1-G1*, but not in those expressing *nef* or *APOL1-G0* (Fig. 3E,F). Nephrocytes expressing *APOL1-G0*+*nef* or *APOL1-G1*+*nef* showed significantly lower LysoTracker signals (Fig. 3E,F). Moreover, *APOL1-G1*+*nef* nephrocytes had the lowest signal (Fig. 3E,F)*.* These findings indicate that HIV-1 Nef exacerbates the changes induced by APOL1-G1 in key endocytic and acidification pathways that are essential for nephrocyte function.

### HIV-1 Nef enhances APOL1-G1-induced autophagic changes, with accumulation of autophagosomes in fly nephrocytes

**Fig. 4.**
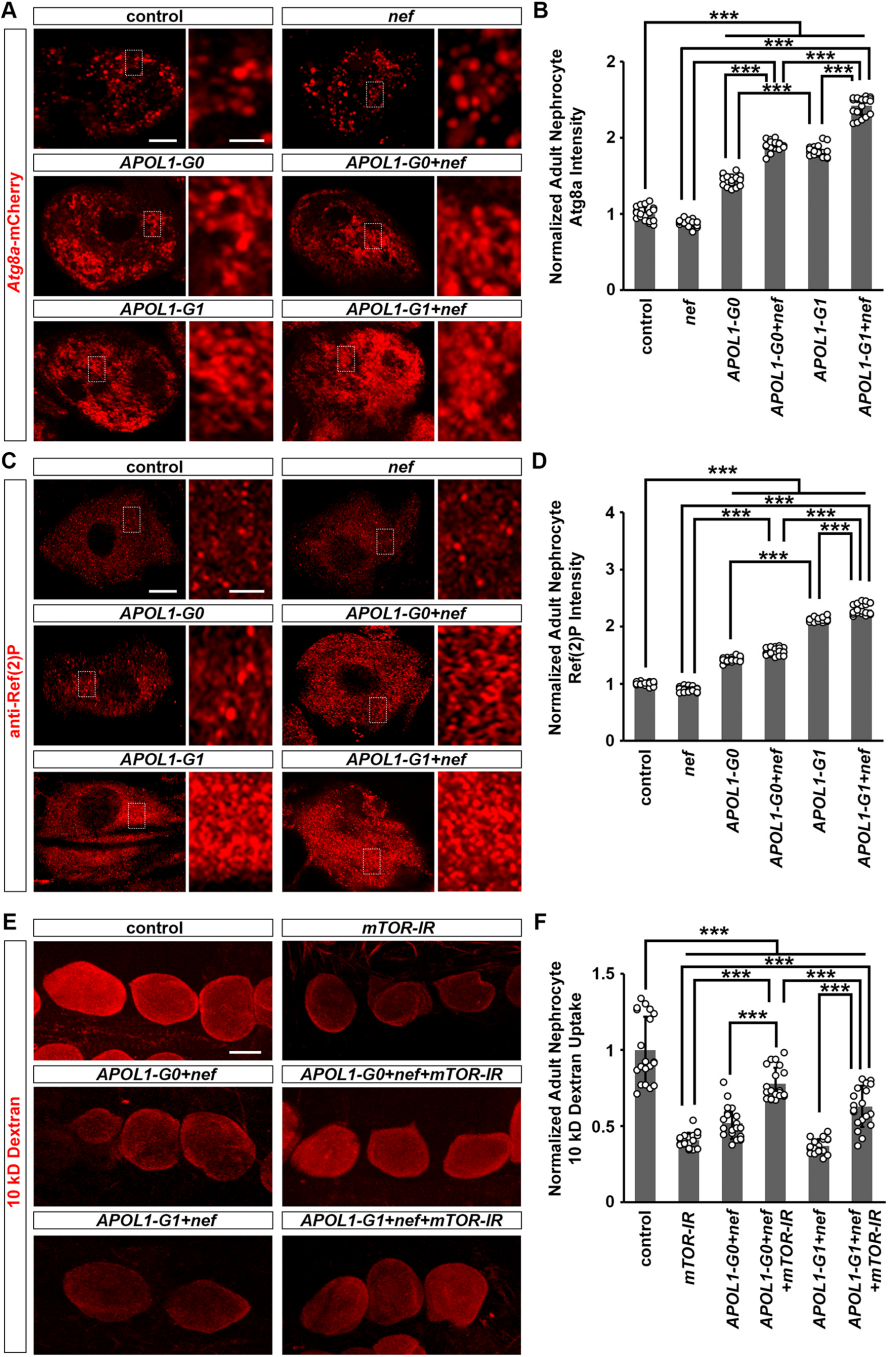
**HIV-1 Nef exacerbated autophagy defects induced by APOL1-G1 in nephrocytes.** (A,B) Flies used (20-day-old adult females): control (*Dot*>*Atg8a*-mCherry); *nef* (*Dot*>*Atg8a*-mCherry*+nef*-OE); *APOL1-G0* (*Dot*>*Atg8a*-mCherry*+APOL1-G0*-OE); *APOL1-G1* (*Dot*>*Atg8a*-mCherry*+APOL1-G1*-OE); *APOL1-G0+nef* (*Dot*>*Atg8a*-mCherry*+APOL1-G0*-OE+*nef*-OE); *APOL1-G1+nef* (*Dot*>*Atg8a*-mCherry *+APOL1-G1*-OE+*nef*-OE). (C,D) Flies used (20-day-old adult females): control (*Dot*>*w*^1118^); *nef* (*Dot*>*nef*-OE); *APOL1-G0* (*Dot*>*APOL1-G0*-OE); *APOL1-G1* (*Dot*>*APOL1-G1*-OE); *APOL1-G0+nef* (*Dot*>*APOL1-G0*-OE+*nef*-OE); *APOL1-G1+nef* (*Dot*>*APOL1-G1*-OE+*nef*-OE). (E,F) Flies used (20-day-old adult females): control (*Dot*>*w*^1118^); *mTor* RNA interference (*mTor*-IR) (*Dot*>*mTor*-IR); *APOL1-G0*+*nef*+*mTor*-IR (*Dot*>*APOL1-G0*-OE+*nef*-OE+*mTor*-IR), *APOL1-G1*+*nef*+*mTor*-IR (*Dot*>*APOL1-G1*-OE+*nef*-OE+*mTor*-IR). (A) Expression of Autophagy-related protein 8a (Atg8a; red, mCherry) in nephrocytes using the nephrocyte-specific driver *Dot*-Gal4 to express HIV-1 *nef* alone or together with *APOL1-G0* and *APOL1-G1*. Boxed areas are shown magnified next to each image. Scale bars: 5 μm; 1 μm (magnified). (B) Quantitation of Atg8a fluorescence intensity, relative to fluorescence in control nephrocytes. *n*=20 flies, per group. (C) Autophagy receptor Refractory to sigma P [Ref(2)P; red] in nephrocytes using the nephrocyte-specific driver *Dot*-Gal4 to express HIV-1 *nef* alone or together with *APOL1-G0* and *APOL1-G1*. Boxed areas are shown magnified next to each image. Scale bars: 5 μm; 1 μm (magnified). (D) Quantitation of Ref(2)P fluorescence intensity, relative to fluorescence in control nephrocytes. *n*=20 flies, per group. (E) 10 kDa fluorescent dextran particle uptake (red) by nephrocytes using nephrocyte-specific driver *Dot*-Gal4 to inhibit *mTor* expression alone or together with *APOL1-G0+nef* and *APOL1-G1+nef* at 22°C. Scale bar: 15 µm. (F) Quantitation of 10 kDa dextran uptake, relative to uptake in control flies. *n*=20 flies, per group. Results are presented as mean±s.d., normalized to the control group. Kruskal–Wallis H-test followed by a Dunn's test; ***P<0.001.

APOL1 can modulate autophagy in various experimental model systems (Fu et al., 2017a; McCarthy et al., 2021; Gerstner et al., 2022; Lee et al., 2023; Chang et al., 2019; Chun et al., 2019; Gatica et al., 2018). In *Drosophila*, Autophagy-related 8a (Atg8a), the equivalent of human LC3 (also known as MAP1LC3A), is a widely used marker of autophagy. Nephrocytes expressing *nef* showed no alteration in Atg8a-mCherry, but those expressing *nef* together with *APOL1* showed significantly higher levels of Atg8a-mCherry, with the highest level of Atg8a found in *APOL1-G1*+*nef*-expressing nephrocytes (Fig. 4A,B). Normally, higher levels of Atg8a indicate increased autophagy (Nagy et al., 2015). However, when we examined the consequence of autophagy using the antibody against Ref(2)P, a marker of cargo destined to be degraded by autophagy (Spitz et al., 2022), we found that Ref(2)P levels actually increased in nephrocytes with *APOL1-G1* or *APOL1-G1*+*nef* expression (Fig. 4C,D), suggesting a reduced level of autophagy. Similar to the Atg8a results, the increase in Ref(2)P level was exacerbated in *APOL-G1*+*nef*-expressing nephrocytes (Fig. 4C,D). To determine whether inhibiting mTor signaling, which can increase autophagy in *Drosophila* nephrocytes (Spitz et al., 2022), could rescue the nephrocyte function decline caused by APOL1 and Nef expression, we expressed *APOL1* and *nef* together with mTor knockdown in *Drosophila* nephrocytes. We observed that *mTor* knockdown alone led to reduced nephrocyte function and decreased cell size. Interestingly, co-expression of *mTor* RNA interference with either *APOL1-G0*+*nef* or *APOL1-G1*+*nef* particularly rescued the nephrocyte functional defects (Fig. 4E,F). These findings suggest that autophagy plays a specific role in mediating the toxicity induced by the combined expression of APOL1 and Nef in nephrocytes.

### HIV-1 Nef and APOL1-G1 reduce autophagy in nephrocytes synergistically

**Fig. 5.**
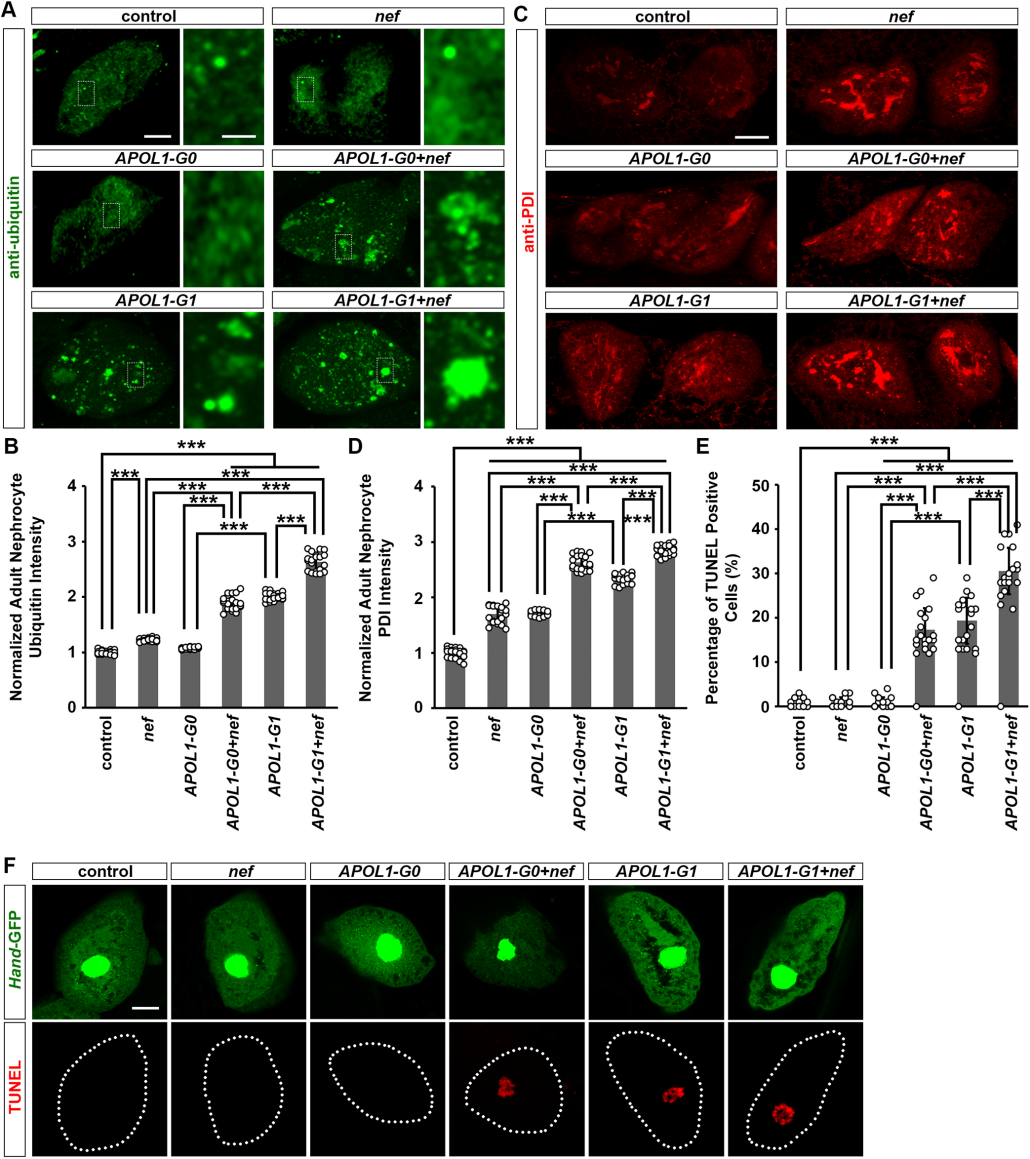
**Expression of HIV-1 protein Nef in nephrocyte-induced ER stress.** (A-D) Flies used (20-day-old adult females): control (*Dot*>*w*^1118^); *nef* (*Dot*>*nef*-OE); *APOL1-G0* (*Dot*>*APOL1-G0*-OE); *APOL1-G1* (*Dot*>*APOL1-G1*-OE); *APOL1-G0+nef* (*Dot*>*APOL1-G0*-OE+*nef*-OE); *APOL1-G1+nef* (*Dot*>*APOL1-G1*-OE+*nef*-OE). (E,F) Flies used (20-day-old adult females): control (*Hand*-GFP, *Dot*>GFP+*w*^1118^); *nef* (*Hand*-GFP, *Dot*>*nef*-OE); *APOL1-G0* (*Hand*-GFP, *Dot*>*APOL1-G0*-OE); *APOL1-G1* (*Hand*-GFP, *Dot*>*APOL1-G1*-OE); *APOL1-G0+nef* (*Hand*-GFP, *Dot*>*APOL1-G0*-OE+*nef*-OE); *APOL1-G1+nef* (*Hand*-GFP, *Dot*>*APOL1-G1*-OE+*nef*-OE). (A) The accumulation of ubiquitinylated proteins was shown by an anti-ubiquitin antibody in nephrocytes with expression of *APOL1-G0* or *APOL1-G1*, with or without HIV-1 *nef*. Boxed areas are shown magnified next to each image. Scale bars: 5 μm; 1 μm (magnified). (B) Quantitation of ubiquitinylated proteins fluorescence intensity, relative to fluorescence in control nephrocytes. *n*=20 flies, per group. (C) The endoplasmic reticulum (ER) stress marker Protein disulfide isomerase (Pdi; red) in nephrocytes using the nephrocyte-specific driver *Dot*-Gal4 to express HIV-1 *nef* alone or together with *APOL1-G0* and *APOL1-G1*. Scale bar: 15 μm. (D) Quantitation of anti-Pdi antibody staining fluorescence intensity, relative to fluorescence in control nephrocytes. *n*=20 flies, per group. (E) Quantitation of percentage of terminal deoxynucleotidyl transferase dUTP nick end labeling (TUNEL)-positive cells in each fly. *n*=20 flies, per group. (F) Apoptosis marker TUNEL (red) in nephrocytes using the nephrocyte-specific driver *Dot*-Gal4 to express HIV-1 *nef* alone or together with *APOL1-G0* and *APOL1-G1*. *Hand*-GFP transgene expression was visualized as green fluorescence concentrated in the nuclei of nephrocytes. Dotted lines indicate the boundary of a cell. Scale bar: 5 μm. Results are presented as mean±s.d., normalized to the control group. Kruskal–Wallis H-test followed by a Dunn's test; ***P<0.001.

Because Ref(2)P levels are a more reliable marker than levels of Atg8a for the end result of autophagy, we hypothesized that the increased Atg8a levels could be due to accumulation of non-functional autophagosome. To verify this hypothesis, we examined the level of ubiquitinylated proteins in nephrocytes using an anti-ubiquitin antibody (Fig. 5A,B) and observed increased accumulation of ubiquitinylated proteins in nephrocytes expressing *APOL1-G1*, but not in those expressing *nef* or *APOL1-G0* (Fig. 5A,B). We also found that *APOL1-G1* and *nef* together induced the highest accumulation of ubiquitinylated proteins in nephrocytes (Fig. 5A,B). This suggests that HIV-1 Nef and APOL1-G1 reduce autophagy in nephrocytes synergistically.

### HIV-1 Nef and APOL1-G1 increase ER stress in nephrocytes synergistically

APOL1-G1 can elicit the ER stress response (Rani and Gautam, 2018; Lee et al., 2023), while HIV-1 Nef can bind the ER chaperone calnexin (CNX; also known as CANX) to induce ER stress (Jennelle et al., 2014; Ryan et al., 2016; Guérin et al., 2008). We previously reported that APOL1-G1 induced ER stress in *Drosophila* nephrocytes (Lee et al., 2023), which is consistent with other studies’ results (Gerstner et al., 2022). Therefore, we assessed Protein disulfide isomerase (Pdi), a marker of ER stress in nephrocytes, using antibody staining. Pdi was significantly increased in nephrocytes expressing *nef* (Fig. 5C,D). This increase was significantly more substantial when *nef* was expressed simultaneously with *APOL1-G1*, even when compared to nephrocytes expressing *APOL1-G1* alone or *APOL1-G0*+*nef* (Fig. 5C,D). However, further studies are needed to define the relative contribution of ER stress to the synergistic effects of APOL1-G1 and HIV-Nef in nephrocytes. Additionally, although we observed that enhancing autophagy rescued the nephrocyte phenotype induced by APOL1-G1 and HIV-Nef (Fig. 4E,F), it is important to note that this effect may be attributed to the broader role of mTor signaling. mTor modulation not only promotes autophagy but also suppresses protein synthesis (Zhao et al., 2015), which could independently alleviate ER stress . Further studies are needed to investigate these mechanisms.

Furthermore, nephrocytes that expressed *APOL1-G1*, but not *nef* or *APOL1-G0*, showed positive results in the terminal deoxynucleotidyl transferase dUTP nick end labeling (TUNEL) assay, a marker of cell death (Fig. 5E). TUNEL-positive cells were also observed when nephrocytes expressed *APOL1-G0*+*nef* or *APOL1-G1*+*nef* (Fig. 5E,F). Additionally, we performed co-labeling of TUNEL and anti-Pyd antibody in *Drosophila* nephrocytes. Because TUNEL marks nuclear DNA fragmentation, the confocal images were taken in the optical medial plane to capture the cell center. In this view, anti-Pyd antibody staining showed a characteristic continuous circular ring in nephrocytes with intact slit diaphragms. In the *APOL1-G0+nef*, *APOL1-G1* and *APOL1-G1+nef* groups, we observed that nephrocytes displaying slit diaphragm defects were also TUNEL positive, indicating these cells were undergoing cell death (Fig. S2). We also observed phenotypic abnormalities in some TUNEL-negative nephrocytes (Fig. S2), suggesting that slit diaphragm damage precedes cell death and that the observed effects are not solely a consequence of apoptosis. These data support the association between slit diaphragm damage and apoptotic processes, indicating that HIV-1 Nef can exacerbate the APOL1-G1-induced autophagy-dependent pathway to further stimulates ER stress, leading to nephrocyte dysfunction and cell death.

## DISCUSSION

**Fig. 6.**
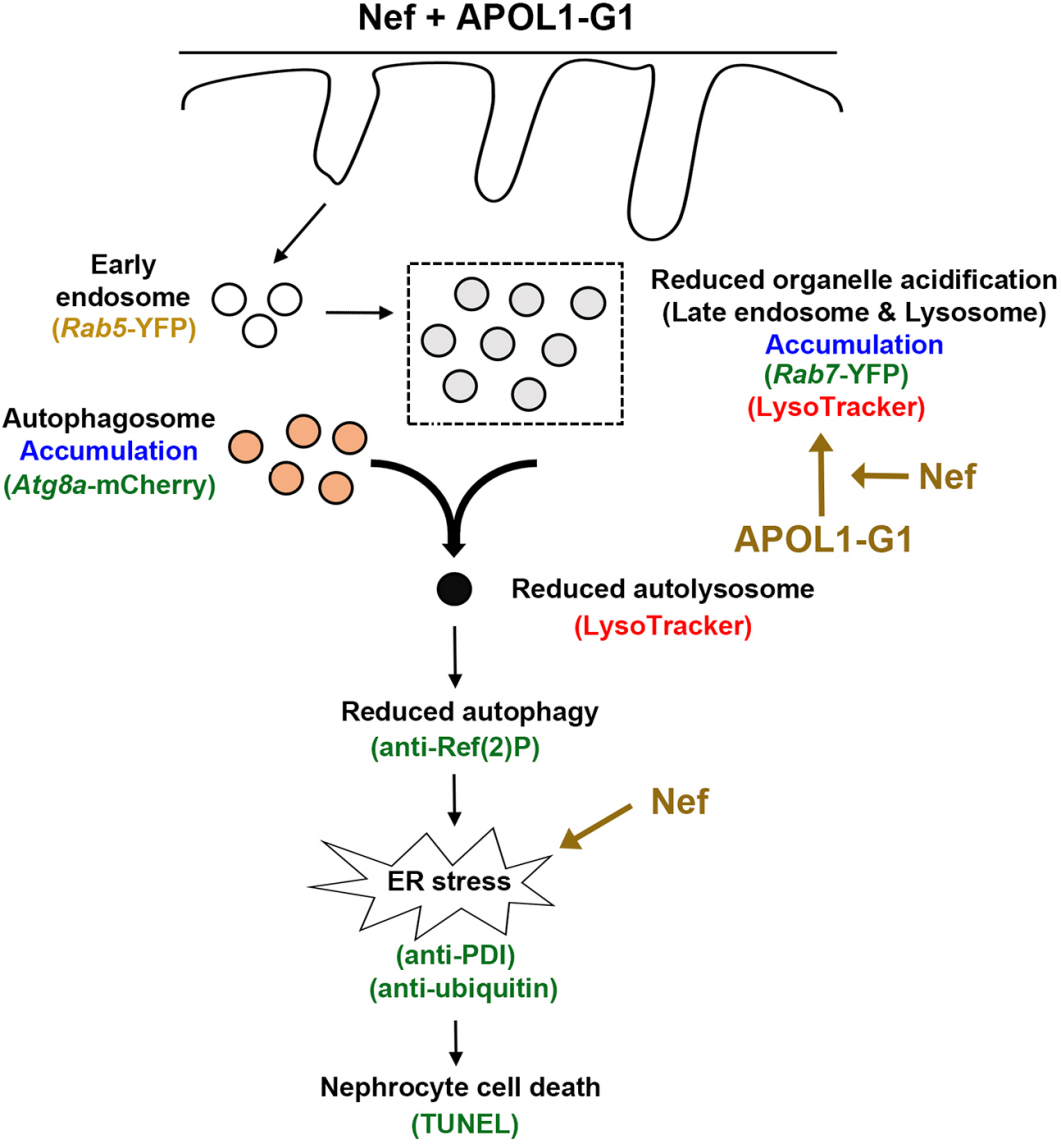
**Model of HIV-1 Nef acting in synergy with APOL1-G1 through ER stress.** Graphic depiction of the proposed model in which Nef exacerbates APOL1-G1-mediated ER stress and causes cellular toxicity. APOL1-G1 causes reduced organelle acidification, which leads to accumulation of non-functional autophagosome and reduced autophagy. Reduced autophagy results in protein accumulation, leading to ER stress. Nef exacerbates the effects of APOL1-G1 on organelle acidification and independently promotes ER stress. Therefore, HIV-1 Nef and APOL1-G1 synergistically increase cellular toxicity through ER stress, which ultimately leads to cell death and tissue damage in the kidneys. (Green text indicates that data showed upregulation, red text indicates that data showed downregulation, yellow text indicates that data showed no change.) APOL1-G1, apolipoprotein L1 risk allele G1; Atg8a-mCherry, Autophagy-related 8a with mCherry fluorescent tag; ER, endoplasmic reticulum; PDI, Protein disulfide isomerase; *Rab5/7*-YFP, *Rab5* or *Rab7* with yellow fluorescent protein tag; Ref(2)P, Refractory to sigma P; TUNEL, terminal deoxynucleotidyl transferase dUTP nick end labeling assay.

We have developed a novel *Drosophila* model to investigate the interaction between HIV-1 Nef and APOL1-G1 in nephrocytes. Our findings demonstrate that HIV-1 Nef significantly worsens the pathogenic effects of APOL1-G1 across various cellular functions, including organelle acidification, autophagy and ER stress. Specifically, APOL1-G1 impairs organelle acidification, leading to the accumulation of non-functional autophagosomes and reduced autophagic activity, which in turn causes protein buildup and subsequent ER stress. Importantly, Nef exacerbates APOL1-G1-induced acidification defects and directly promotes ER stress, creating a synergistic increase in cellular toxicity. This combined stress ultimately leads to cell death and tissue damage in kidney cells, as demonstrated in Fig. 6. However, owing to technical limitations, determining the subcellular localization of *APOL1* and *nef* transgenes in *Drosophila* nephrocytes remains challenging, limiting our ability to gain further insights. Here, we were unable to determine whether Nef affects the subcellular localization of APOL1, and further studies are underway to resolve this issue.

APOL1-G1-induced cytotoxicity is not exclusively dependent on the nucleotide substitutions that define this risk variant. It is also affected by the BH3 domain (Gerstner et al., 2022; Heneghan et al., 2015), exon 4-encoded sequences (Chang et al., 2019) and different haplotypes of *APOL1* (Lannon et al., 2019). Therefore, we generated Tg flies expressing a common natural *APOL1-G1* derived from culture podocytes from a child with HIVAN (Xie et al., 2014). This *APOL1-G1* contains the haplotypes E150, I228 and K255, and only differs at S342 and I384 from a common natural *APOL1-G0* haplotype used to generate the control flies. Notably, this *APOL1-G0* control haplotype is more toxic in cultured human kidney epithelial cells (HEK 293) than the reference *APOL1-G0* haplotype (Xie et al., 2014; O'Toole et al., 2018; Kruzel-Davila et al., 2017; Lan et al., 2014; Nichols et al., 2015; Olabisi et al., 2016; Granado et al., 2017; Hayek et al., 2017; Ma et al., 2017; Wen et al., 2018). We previously showed that *APOL1-G0* expression in nephrocytes induced toxicity, albeit less severe compared to that induced by *APOL1-G1* expression (O'Toole et al., 2018; Lee et al., 2023). When we reduced the expression levels of *APOL1-G0* in nephrocytes by lowering the temperature to 22°C, *APOL1-G0* toxicity was eliminated (Han and Olson, 2005); hence, all fly experiments in the current study were conducted at 22°C (instead of 25°C used in our previous study) so that only *APOL1-G1* remains toxic. Although nephrocyte function appears to be unaffected in *APOL1-G0*-expressing flies at 22°C, the downstream pathway may still be impacted. Nevertheless, we found that Nef could also synergize with APOL1-G0, making it toxic at 22°C (Fig. 1), consistent with clinical studies showing that people of West African genetic ancestry living with a high HIV viral load can develop HIVAN even if they do not carry an *APOL1-RA* (Kasembeli et al., 2015).

A study using nephrin promoter-driven *APOL1-G0* Tg mice crossbred with HIV-Tg26 mice showed that fewer renal HIVAN lesions were developed compared to single HIV-Tg26 mice, suggesting a protective role for APOL1-G0 against HIV-induced nephropathy (Zhang et al., 2013). However, studies in *APOL1* Tg mice with a bacterial artificial chromosome (BAC) harboring human *APOL1-G0*, *-G1* or *-G2* found greater renal pathogenicity in *G2*/*G2* Tg mice, compared to *G1*/*G1* Tg mice, and that *APOL1-G0* did not rescue *APOL1-RA*-induced kidney injury when these mice were injected with interferon-gamma (INF-γ) (Bruggeman et al., 2019). Likewise, we found that *APOL1-G2* Tg flies developed more significant nephrocyte injury compared to *APOL1-G1* Tg flies (Lee et al., 2023). Overall, these studies indicate that the toxicity of APOL1-RA is mainly a function of the renal expression level (Bruggeman et al., 2019), explaining why *APOL1* Tg mice driven by different promoters show different results (Yoshida et al., 2023; Beckerman et al., 2017; McCarthy et al., 2021; Bruggeman et al., 2016). Our data are also consistent with a recent study using dual BAC/*APOL1*-HIV-Tg26 mice (Yoshida et al., 2024) to show that expression of *APOL1-G0* did not minimize HIVAN lesions and expression of *APOL1-G1* aggravated them (Yoshida et al., 2024).

Previous studies showed that HIV-1 Nef can induce ER stress by binding a chaperone protein, CNX (Jennelle et al., 2014; Ryan et al., 2016; Guérin et al., 2008). We showed that Nef caused ER stress in nephrocytes through a different pathway. We demonstrated that Nef, together with APOL1-G1, disrupted the autophagy pathway (Fig. 4), promoting additional ER stress (Rani and Gautam, 2018; Lee et al., 2023) (Fig. 5). Defective endosomal trafficking has been reported in yeast and flies expressing *APOL1-G1* (Gerstner et al., 2022). Likewise, immunofluorescence studies in cultured human podocytes revealed increased RAB7 (late endosomes)- and LC3II (autophagosomes)-positive organelles in cells expressing the high-risk (*G1*/*G2*) compared to low-risk (*G0*/*G0*) *APOL1* alleles (Fu et al., 2017a). Studies in inducible podocyte-specific *APOL1-RA* transgenic mice also showed that APOL1-G1 interferes with intracellular vesicular trafficking by impairing endocytic, autophagic and acidification pathways, as well as by disrupting the maturation of autophagosomes and autophagic flux, which correlated with the development of FSGS (Fu et al., 2017a). Expression of *APOL1-RA* was also shown to affect autophagy in cultured human podocytes (Kumar et al., 2019; Ekulu et al., 2021).

Several studies have shown that HIV-1 can restrict autophagy to promote viral replication by inhibiting the fusion between autophagosomes and lysosomes (Chang et al., 2019; Campbell et al., 2015; Saribas et al., 2015; Gupta et al., 2017). Nef overexpression in human astrocytes led to increased ATG8/LC3 (Saribas et al., 2015), and impaired acidification in human cardiomyocytes, altering RAB7 localization and autophagy pathways (Gupta et al., 2017). Alternatively, HIV-1 Nef reduced the fusion of autophagosomes to lysosomes by interacting with beclin1 (BECN1)/RAB7 in cultured human cells (Franco-Juárez et al., 2022; Zhang et al., 2020). Together, these and our findings demonstrate that HIV-1 Nef-mediated inhibition of autophagic flux is conserved across cell types and species. Notably, the minor changes induced by Nef alone in nephrocytes resemble the mild podocyte changes observed in Tg mice with podocyte-specific *nef* expression (Zuo et al., 2006; Husain et al., 2005). However, other studies showed more severe podocyte injury when HIV-1 *nef* and *vpr* were expressed simultaneously in mouse podocytes (Zuo et al., 2006), suggesting that other HIV-1 genes and cytokines released by HIV-infected cells are needed to fully evoke the HIVAN phenotype. HIV-*nef* Tg mice driven by the CD4 promoter also developed kidney disease (Hu et al., 2023; Hanna et al., 1998a,b). Even though podocytes might not express HIV-1 genes (Hu et al., 2023; Hanna et al., 1998a,b), Nef can still be released by HIV-infected cells predominately in exosomes (Dubrovsky et al., 2022) and can be taken up by uninfected cells (Mukhamedova et al., 2019).

Our fly model has limitations, including that it is based on the ectopic overexpression of *APOL1* haplotypes, which can affect the intracellular localization and cytotoxicity of APOL1 (Baumann et al., 2004; Li et al., 2017), and bypass the infection of renal cells and the effects of other HIV genes and circulating cytokines acting as additional risk factors. HIVAN is a complex disease involving many renal cell types and structures that cannot be studied in flies. However, our model permits the exploration of HIV-1 Nef and APOL1 interactions independently of many confounding variables. Most findings from Tg mice, cultured podocytes and HIV-infected cells published so far are consistent with what we observed in nephrocytes. Because most relevant pathogenic pathways are highly conserved between fly nephrocytes and human podocytes, this new fly model provides a unique opportunity for rapid and cost-effective genetic or drug screens to better understand the interactions between APOL1 and HIV-1 for inducing renal injuries, as well as to discover novel therapeutics.

*Drosophila* is evolutionarily distant from humans, and we have accordingly discussed the limitations of using this model to study HIV-associated kidney diseases. However, because HIV-1 only infects human cells and rodents do not express APOL1, there are currently no ideal animal models to study the interaction between APOL1 and HIV-Nef in podocytes. Notably, prior studies using dual APOL1-HIV Tg mice were unable to determine whether APOL1-G1 and HIV-Nef interact specifically in mouse podocytes *in vivo* (Yoshida et al., 2024). In contrast, *Drosophila* nephrocytes provide a unique and cost-effective *in vivo* model system to explore how APOL1-G1 and HIV-Nef may interact.

Supporting the relevance of this model, our previous findings using nephrocyte-specific *APOL1-G1*-expressing flies (Fu et al., 2017a) and *APOL1-G2*-expressing flies (Zhu et al., 2023) have been validated by independent studies in *APOL1* Tg mice and cultured mouse and human podocytes (Beckerman et al., 2017; Blazer et al. 2022). Moreover, *Drosophila* models of APOL1 have been adopted by other groups to uncover key aspects of APOL1 renal toxicity mechanisms (Kruzel-Davila et al., 2017; Gerstner et al., 2022). Thus, despite the evolutionary distance between *Drosophila* and human, it is now well accepted that *Drosophila* serves as a valuable model system to study the mechanisms of APOL1-associated renal toxicity.

## MATERIALS AND METHODS

### Fly strains

Flies were maintained on standard food (Meidi LLC) at 22°C under standard conditions. The following *Drosophila* lines were obtained from the Bloomington *Drosophila* Stock Center (BDSC): *Dot*-Gal4 (ID_6903), UAS-YFP-*Rab5* (ID_24616), UAS-YFP-*Rab7* (ID_23270), UAS-mCherry-*Atg8a* (ID_37750), UAS-GFP (ID_32184), UAS-*mTor*-IR (ID_33951 and 34639) and *w*1118 (ID_3605). We previously generated the *Hand*-GFP flies labeling the nuclei of nephrocytes and cardiomyocytes (Murakawa et al., 1988). When comparing the *Drosophila* nephrocyte functional or morphological differences between *Dot*>*w^11^18* and *Dot*>UAS-GFP, we observed no significant differences between these two groups (Fig. S1). Additionally, we compared *Dot*>UAS-*APOL1-G1* and *Dot*>UAS-*APOL1-G1*+UAS-GFP, observing that *Dot*>UAS-*APOL1-G1*+UAS-GFP exhibited a similar phenotype to *Dot*>UAS-*APOL1-G1*, with nephrocyte functional decline and increased cell size (Fig. S1). Based on this, we used *Dot*>*w^1118^* as a control.

The *APOL1-G0*- and *APOL1-G1* (cDNA from a child with HIVAN)-expressing *Drosophila* lines were generated as described previously (O'Toole et al., 2018). HIV-1 *nef* cDNA (pGM91) was obtained from the National Institutes of Health AIDS Research and Reference Reagent Program (contributed by Dr John Rossi) (Schneider et al., 2012). To generate UAS-*nef* constructs, the *nef* allele cDNA was cloned into pUAST-attB, and the transgene was introduced into a docking site by germline transformation. Flies expressing *Dot*-Gal4;UAS-*APOL1-*(*G0*/*G1*) were crossed with UAS-*nef* transgenic flies at 22°C. All the crosses were repeated three times independently.

### 10 kDa dextran and FITC-albumin uptake assay

Dextran and albumin uptake by nephrocytes was assessed *ex vivo* in (20-day-old) female adult flies to expose nephrocytes to artificial hemolymph containing 70 mmol/l NaCl, 5 mmol/l KCl, 1.5 mmol/l CaCl_2_, 4 mmol/l MgCl_2_, 10 mmol/l NaHCO_3,_ 5 mmol/l trehalose, 115 mmol/l sucrose and 5 mmol/l HEPES (Sigma-Aldrich). Following a 20-min incubation with 10 kDa Texas Red-dextran (0.05 mg/ml, Invitrogen) or FITC-albumin (100 mM, Sigma-Aldrich) and fixation (10 min) in 4% paraformaldehyde in phosphate buffered saline (4% PFA), the nephrocytes were mounted with VectaShield mounting medium (Vector Laboratories) and imaged.

### Nephrocyte size and number

Nephrocytes were dissected from 20-day-old female adult flies and kept in artificial hemolymph, followed by fixation (10 min) in 4% PFA, and imaged. Nephrocyte size was determined using the area measurement function in ImageJ (Schneider et al., 2012) (version 1.52a). Nephrocyte numbers were manually counted using images of *Hand*-GFP labeling.

### Immunochemistry

Female (20-day-old) flies were dissected and fixed [20 s heat fix in 100°C artificial hemolymph for anti-Pyd antibody or in 4% PFA for anti-Ref(2)P, anti-ubiquitin (FK2) and anti-Pdi antibodies]. Immunochemistry was carried out using established methods (O'Toole et al., 2018). Antibodies used in this study include mouse anti-Pyd antibody [1:100; Developmental Studies Hybridoma Bank (DSHB)], rabbit anti-Ref(2)P (1:100; ab178440, Abcam), mouse anti-ubiquitin FK2 (1:100; BWL-PW8810, Enzo Life Sciences), rabbit anti-Pdi (1:100; ZRB1846-25UL, Sigma-Aldrich) and Alexa Fluor 555 (1:1000; Thermo Fisher Scientific).

### LysoTracker assay

Fly nephrocytes were dissected in artificial hemolymph, incubated (20 min) with LysoTracker (Thermo Fisher Scientific), fixed (10 min) with 4% PFA, mounted with VectaShield and imaged.

### TUNEL assay

Nephrocytes were dissected in artificial hemolymph and heat fixed (100°C, 20 min) in 4% PFA, blocked with blocking buffer (50 mM Tris-HCl pH 7.4, 0.1% Triton X-100 and 188 mM NaCl), incubated with an In Situ Cell Death Detection Kit (Roche), mounted with VectaShield and imaged.

### Confocal imaging

Confocal images were obtained using a ZEISS LSM900 microscope and ZEN Blue (edition 3.0) acquisition software. For quantitative comparison of intensities, common settings were chosen to avoid oversaturation and then applied across images for all samples within an assay. ImageJ (Schneider et al., 2012) (version 1.52a) was used for all image processing. For quantification, *Rab5*-YFP fluorescence intensity was measured in cortical areas, and *Rab7*-YFP and Pdi fluorescence intensities were measured in whole cells.

### Statistical analysis

Statistical tests were performed using PAST.exe software (University of Oslo). Data were tested for normality using the Shapiro–Wilk test (α=0.05). Normally distributed data were analyzed by one-way ANOVA followed by Tukey–Kramer post-test for comparing multiple groups. Non-normal distributed data were analyzed by Kruskal–Wallis H-test followed by a Dunn's test for comparisons between multiple groups. Results are presented as mean±s.d. Statistical significance was determined as *P*<0.05. Details are provided in Dataset 1.

## Supplementary Material

10.1242/dmm.052178_sup1 Supplementary information

Dataset 1. Statistics.
