# Supplementary material for: HIV-1 Nef synergizes with APOL1-G1 to induce nephrocyte cell death in HIV-related kidney diseases
Source: Dis Model Mech. 2025 Aug 1;18(7):dmm052178. doi: 10.1242/dmm.052178 (PMC12352291; doi:10.1242/dmm.052178)
Supplement: Supplementary information [file dmm-18-052178-s1.pdf]

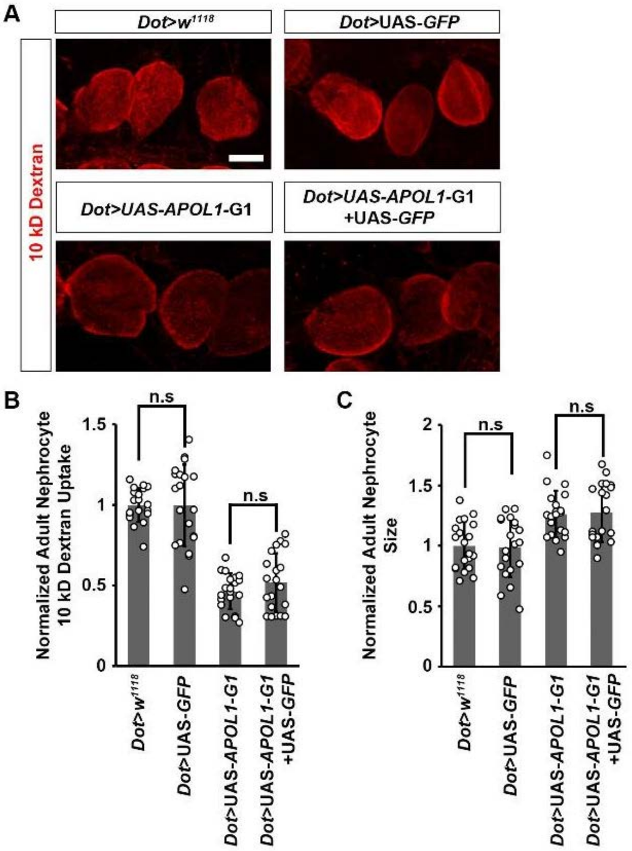

Fig. S1. Expression of green fluorescence protein (GFP) did not cause additional nephrocyte functional and morphological defects. (A) 10kD fluorescent dextran particle uptake (red) by nephrocytes using nephrocyte specific driver *Dot*-Gal4 to express GFP alone or together with *APOL1-G1* at 22°C. Scale bar: 15 µm. **(B)** Quantitation of 10kD dextran uptake, relative to uptake in control flies. n=20 flies, per group. **(C)** Quantitation of adult nephrocyte size, relative to size in control flies. n=20 flies, per group. Results have been presented as mean ± s.d., normalized to the control group. Kruskal–Wallis H-test followed by a Dunn's test; statistical significance: *P<0.05, **P<0.01, ***P<0.001.

Disease Models & Mechanisms • Supplementary information

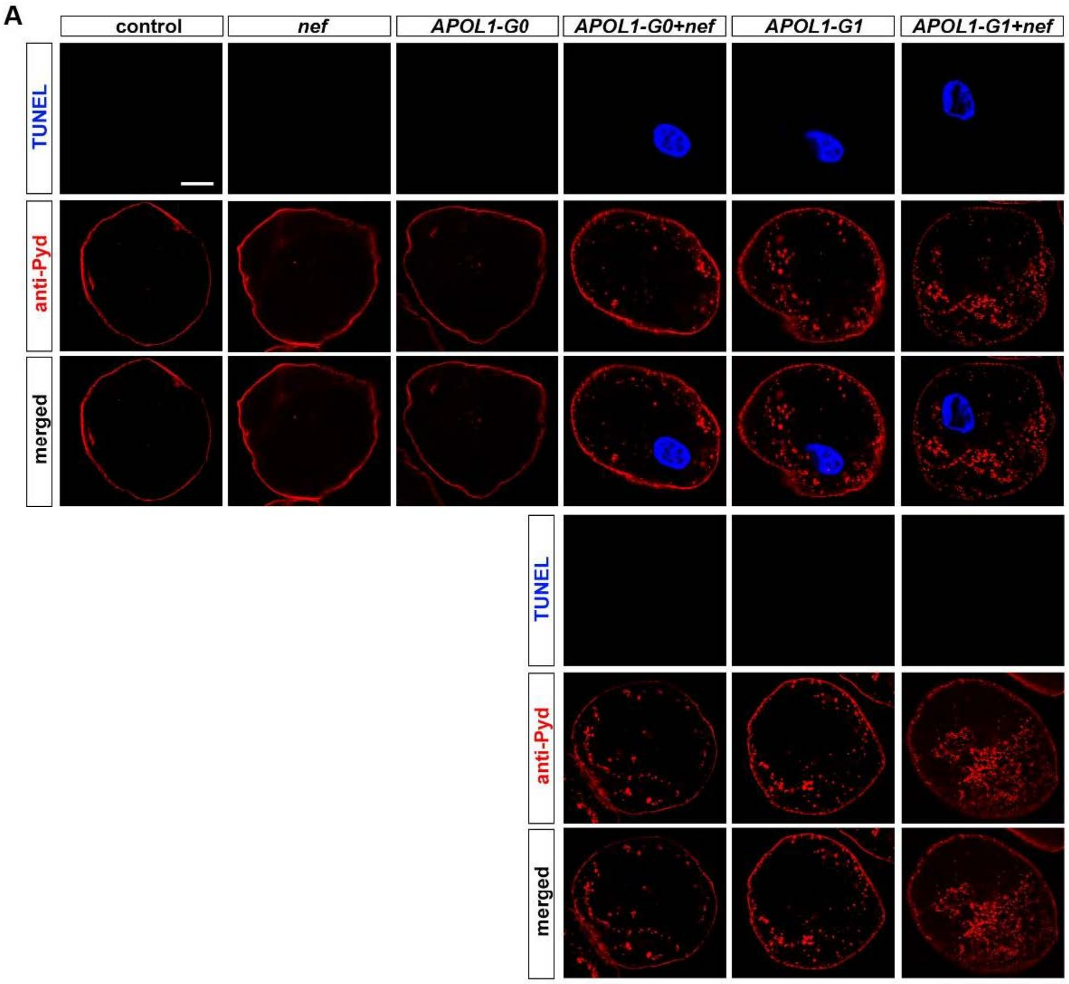

Fig. S2. Association between slit diaphragm damage and apoptotic processes. (A) Apoptosis marker TUNEL (blue) co-labeled with slit diaphragm protein Polychaetoid (Pyd; red) at the medial optical sections in nephrocytes using the nephrocyte specific driver *Dot*-Gal4 to express HIV-1 *nef* alone or together with *APOL1-G0* and *APOL1-G1*. Scale bar: 5 μm.

Dataset 1. Statistics.

Available for download at
https://journals.biologists.com/dmm/article-lookup/doi/10.1242/dmm.052178#supplementary-data
